# Microscopic observations of SARS‐CoV‐2 like particles in different oral samples

**DOI:** 10.1111/eos.12903

**Published:** 2022-11-20

**Authors:** Djamal Brahim Belhaouari, Jean‐Pierre Baudoin, Jean‐Christophe Lagier, Virginie Monnet‐Corti, Bernard La Scola, Angéline Antezack

**Affiliations:** ^1^ IRD, AP‐HM, IHU Méditerranée Infection, MEPHI Aix Marseille Univ Marseille France; ^2^ Assistance Publique‐Hopitaux de Marseille Hopital Timone Marseille France; ^3^ Faculté des Sciences Médicales et Paramédicales Ecole de Médecine Dentaire Aix Marseille Univ Marseille France; ^4^ Department of Veterinary Pathobiology, College of Veterinary Medicine & Biomedical Sciences Texas A&M University College Station TX USA

**Keywords:** COVID‐19, dental plaque, saliva, scanning electron microscopy, virology

## Abstract

The emerging coronavirus pneumonia epidemic caused by the SARS‐CoV‐2 infection has spread rapidly around the world. The main routes of transmission of SARS‐CoV‐2 are currently recognised as aerosol/droplet inhalation. However, the involvement of the oral cavity in coronavirus disease 2019 (COVID‐19) is poorly known. The current data indicates the presence of viral RNA in oral samples, suggesting the implication of saliva in SARS‐CoV‐2 transmission, however, no direct observation of SARS‐CoV‐2 particles in different oral samples has been reported. In this study, we investigated whether particles of SARS‐CoV‐2 were present in oral samples collected from three symptomatic COVID‐19 patients. Using scanning electron microscopy (SEM), the correlative strategy of light microscopy and electron microscopy and immunofluorescence staining, we showed the presence of SARS‐like particles in RT‐qPCR SARS‐CoV‐2‐positive saliva, dental plaque and gingival crevicular fluid (GCF) samples. In the saliva samples, we demonstrated the presence of epithelial oral cells with morphogenetic features of SARS‐CoV‐2 infected cells. Inside those cells, vacuoles filled with nascent particles were observed, suggesting the potential infection and replication of SARS‐CoV‐2 in oral tissues. Our results corroborate previous studies and confirm that the oral cavity may be a potential niche for SARS‐CoV‐2 infection and a potential source of transmission.

## INTRODUCTION

At the end of 2019, SARS‐CoV‐2 emerged in the city of Wuhan, China, and led to an outbreak of severe acute respiratory syndrome (COVID‐19) [1].

As of 3rd December 2021, a total of 263,563,622 confirmed cases of COVID‐19, including 5,232,562 deaths, have been observed globally [2]. SARS‐CoV‐2 is an enveloped, positive sense, single‐stranded RNA virus that belongs to the family *Coronaviridae* [3]. This virus is highly transmissible and presents a broad tissue tropism that promotes rapid human‐to‐human transmission and intercontinental spread [4].

The involvement of the oral cavity in COVID‐19 is not well understood. A dry mouth, dysgeusia, changes in tongue sensations, ulceration, swelling, muscle pain during mastication, and herpetic lesions have been described as common COVID‐19 oral symptoms [5]. These symptoms most often appear after general symptoms, such as a fever, cough and asthenia. However, they may still be the initial or only sign of COVID‐19, and subjects with negative SARS‐CoV‐2 respiratory swabs may have positive salivary samples at the same time [6].

SARS‐CoV‐2 uses cellular factors, such as angiotensin‐converting enzyme 2 (ACE2) and the transmembrane protease serine 2 (TMPRSS2), to infect host cells [7]. The expression of both ACE2 and TMPRSS2 has been reported in human oral tissue, suggesting that the oral cavity may be considered a major entry point for the virus [8, 9]. Huang et al. [10] recently reported that salivary glands could host replicating SARS‐CoV‐2 by detecting replication strand (sense) RNA using in situ hybridization, which suggests that SARS‐Cov‐2 might primarily infect cells in situ before being shed into the saliva. In addition, using in situ hybridization and 3D confocal microscopy, the authors observed that saliva from symptomatic COVID‐19 subjects harbored epithelial cells with ACE2 and TMPRSS2 expression, of which approximately 5%−10% were infected with SARS‐CoV‐2 [10]. These findings suggest that expelled oral droplets containing infectious virus and infected cells might be a source of airborne transmission of SARS‐CoV‐2.

Dental plaque and gingival crevicular fluid (GCF) have also been explored as possible diagnostic oral samples in two studies using real‐time quantitative polymerase chain reaction (RT‐qPCR). In a first study, Gomes et al. [11] found that 18.6% of symptomatic subjects whose nasopharyngeal and oropharyngeal samples tested positive harbored SARS‐CoV‐2 RNA in their dental plaque. In a second study, Gupta et al. [12] detected SARS‐CoV‐2 RNA in 63.6% of GCF samples from COVID‐19 positive patients tested by nasopharyngeal swab. To our knowledge, no isolation or direct observation of SARS‐CoV‐2 virions particles has been reported from GCF and dental plaque samples.

The aim of this study was to investigate the presence of SARS‐CoV‐2 particles in different oral samples by using scanning electron microscopy (SEM) and correlative light‐electron‐microscopy.

## MATERIAL AND METHODS

### Study participants

The study was carried out by the Institut Hospitalo‐Universitaire Méditerranée Infection, Assistance Publique‐Hôpitaux de Marseille, France, in collaboration with the Department of Periodontology at Aix‐Marseille University, Marseille, France. The study was approved by the Comité de Protection des Personnes Sud‐Ouest et Outre‐Mer 1 (n ID RCB: 2020‐A01234‐35—CPP 1‐20‐075 ID9806) as part of the mouth microbiota inventory. The study participants and a negative control participant were recruited from patients presenting to the Institut Hospitalo‐Universitaire Méditerranée Infection after their COVID‐19 status was confirmed by nasopharyngeal swab testing. Informed consent was obtained from each patient. Epidemiological data were recorded, and a periodontal examination was performed by the same periodontist. Patient data are summarized in Table 1.

**TABLE 1:** Epidemiological data of the three patients included in the study (patient 1–3) and the negative control (patient 4)

**Patient**	**Age (years)**	**Sex**	**Underlying systemic condition**	**Oral status**	**COVID‐19 status**	**Ct value of *E* gene in nasopharyngeal swab** ^a^
1	57	Male	–	Gingivitis	Symptomatic	24.0
2	65	Male	Diabetes, Crohn's disease	Periodontitis stage III grade C	Symptomatic	23.4
3	81	Female	Hypertension, hypothyroidism	Totally edentulous	Symptomatic	19.9
4	29	Female	–	Healthy periodontium	–	>35

^a^One swab for both nares.

### Sample collection

All study participants were asked to not eat, drink or brush their teeth 1 h before sample collection. Samples were collected by the same periodontist with appropriate protective measures in accordance with the institute's guidelines. For unstimulated whole saliva, each patient was asked to expectorate the whole saliva into a 50 ml centrifuge tube until a minimum saliva volume of 2 ml was collected. For dental plaque, a supragingival dental plaque from the dentate patients was collected using a sterile curette and placed into a 1.5 ml Eppendorf tube. For GCF, all supra‐gingival plaque facing the sampling area was removed with a sterile curette to avoid contaminating the samples. The site was then isolated and dried with cotton rolls. GCF was collected using sterile absorbent paper points (Paper Points No.20, VDW‐Zipperer1) carefully positioned into the periodontal pocket or the gingival sulcus and left for 30 s. GCF within the periodontal pocket or the sulcus was absorbed by the paper points through capillary action. A total of six paper points were obtained from the dentate patients and placed into a 1.5‐ml Eppendorf tube. The samples were immediately stored at −80°C.

### Reverse transcription—polymerase chain reaction

The extraction of viral nucleic acids (RNA) was performed using the EZ1 Virus Mini Kit (Qiagen), following the recommended procedures. The procedure for Reverse Transcription—Polymerase Chain Reaction (RT‐PCR) targeting the SARS‐CoV‐2 *E* gene has been detailed elsewhere [13].

### Fast track diagnosis (FTD) respiratory pathogens 21 assay

For the detection of viruses and bacteria potentially present in the samples, multiplex real‐time reverse transcription (RT)‐PCR was used with the FTD Respiratory pathogens 21 kit (Fast Track Diagnosis). This assay allows the concurrent detection of 17 viruses and one bacteria using five tubes containing primer and probe mix preparation: tube‐1 (Influenza virus A, Influenza virus A subtype H1N1 [Pandemic H1N1], Human rhinoviruses, Influenza virus B); tube‐2 (Human coronaviruses NL63, 229E, OC43 and HKU1); tube‐3 (HPIV‐2, 3 and 4 and internal control), Tube‐4 (Human papillomavirus‐1, Mycoplasma pneumoniae, Human bocavirus, Human metapneumovirus); and Tube‐5 (Respiratory Syncytial virus A and B, Human adenovirus, enteroviruses and Human parechovirus). The multiplex real‐time reverse transcription (RT)‐PCR thermal profile for the FTD kit was as follows: 15 min at 50°C, 10 min at 95°C, then 40 cycles including 8 s at 95°C and 34 s at 60°C.

### Resin embedding and ultramicrotomy for SEM of GCF and immunohistochemistry and correlative light and electron microscopy of dental plaque

The dental plaque and GCF paper point samples were fixed with 4% paraformaldehyde in 0.1‐M sodium cacodylate buffer for 5 h at 4°C. Resin embedding was microwave‐assisted with a PELCO BiowavePro+ (Ted Pella). After rinsing twice with a mixture of 0.2‐M saccharose/0.1‐M sodium cacodylate and once with distilled water, samples were gradually dehydrated by successive baths in 50%, 70% and 96% ethanol. Substitution with medium grade LR–White resin (Polysciences) was achieved by two incubations with a mixture of 100% LR–White resin and 96% ethanol in a 2:1 ratio, two incubations with 100% LR–White resin, and completed with samples in 100% LR–White resin. Resin heat‐curing was achieved by polymerization for 72 h at 60°C. All solutions used above were 0.2‐μm filtered. Ultrathin 100 nm sections were cut using a UC7 ultramicrotome (Leica Microsystems) and placed on HR25 300 Mesh Copper/Rhodium grids (TAAB).

### Immunohistochemistry for immunohistochemistry and correlative light and electron microscopy of dental plaque

For anti‐SARS‐CoV‐2 immunohistochemistry, we used a permeabilization‐free procedure. Ultrathin sections of dental plaque samples were incubated for 30 min with bovine serum albumin (BSA) 0.1% in H_2_O, with rabbit IgG anti‐SARS‐CoV‐2 primary antibody (Ref. PA5‐81795; Thermo‐Fischer Scientific) at 1/1000 dilution in H_2_O for 3 h, washed twice with BSA 0.1% in H_2_O for 5 min, incubated with secondary anti‐rabbit Alexa‐647 or secondary antibody Alexa‐555 (Ref. A32733; Thermo‐Fischer Scientific) at 1/100 dilution in H_2_O for 45 min, and washed for 5 min with BSA 0.1% in H_2_O. Sections were also stained with Hoechst3342 (Ref. 62249, Thermo‐Fisher Scientific) at 1/1000 dilution in H_2_O for 30 min. Finally, sections were washed in H_2_O and air‐dried. For all steps above, incubation took place at room temperature and grids with sections facing down were successively placed on 30 μl drops of respective solutions in a humidified chamber.

### Confocal laser scanning microscopy for immunohistochemistry and correlative light and electron microscopy of dental plaque

Electron microscopy grids were placed on a thin glass slide with sections facing down and imaged using confocal laser scanning microscopy on an inverted LSM800 (Zeiss) microscope. Acquisitions were performed with ×40 objective and a zoom between 0.5 and 1.7. For the Hoechst3342 stain, Alexa‐555 and Alexa‐647 fluorophores imaging, 405 nm, 556 nm and 640 nm lasers were used, respectively. Maximal Z‐projections (mean thickness 8 μm) were used to reconstruct a signal from the whole sections thickness. The image size was 1024 × 1024 pixels.

### Immunocytochemistry of saliva samples

Saliva samples were fixed with 4% paraformaldehyde for 20 min and stored in phosphate buffered saline (PBS) at 4°C. Saliva samples were then concentrated and deposited on microscopy slides using cytospin. Fixed samples were permeabilized by incubation with 0.1% Triton X‐100 for 5 min, blocked by incubation for 30 min with 5% normal goat serum in PBS, and incubated with primary anti‐SARS‐CoV‐2 antibody (Ref. PA5‐81795; Thermo‐Fischer Scientific) at 1/1000 dilution for 3 h at 28°C in a humidified chamber. Cells were washed three times with 0.1% Triton ×100 in PBS and then incubated for 1 h with a secondary anti‐rabbit Alexa‐647 antibody at 1/100 dilution. Coverslips were then rinsed four times with PBS. DNA was stained using Hoechst3342 (Ref. 62249, Thermo‐Fisher Scientific), with 30 min of incubation at room temperature, and two washes with PBS and one with distilled water. Coverslips were mounted on glass slides and imaged with a LSM800 microscope (Zeiss).

### Resin embedding and ultramicrotomy of saliva samples

Saliva samples were fixed with glutaraldehyde (2.5%) in a 0.1 M sodium cacodylate buffer for 12 h at 4°C. Saliva samples were deposited in Greiner Bio‐One 96‐well single‐break strip microplates (Greiner Bio‐One) coated with poly‐L lysine to retain the material. Resin embedding of the microplates was then performed as previously described [14]. Resin embedding was microwave‐assisted with PELCO BiowavePro+ (Ted Pella). Samples were washed with a mixture of 0.2 M saccharose/0.1 M sodium cacodylate and post‐fixed with 1% OsO_4_ diluted in 0.2 M potassium hexa‐cyanoferrate (III)/0.1 251 M sodium cacodylate buffer. After being washed with distilled water, samples were gradually dehydrated by successive baths containing 30% to 100% ethanol. Substitution with Epon resin was achieved by incubations with 25%–100% Epon resin, and samples were placed in a polymerization chamber. Resin microwave‐curing was performed for a total of 2 h. After curing, the resin blocks were manually trimmed with a razor blade and the bases of the dishes were detached by cold shock via immersion in liquid nitrogen for 20 s. Resin blocks were placed in a UC7 ultramicrotome (Leica), trimmed to pyramids, and ultrathin 100 nm sections were cut and placed on HR25 300 Mesh Copper/Rhodium grids (TAAB).

### Scanning electron microscopy (SEM)

Ultra‐thin sections were contrasted with uranyl acetate and lead citrate according to Reynolds [15]. Grids were attached with double‐sided tape to a glass slide and platinum‐coated at 10 mA for 20 s with a MC1000 sputter coater (Hitachi High‐Technologies). Electron micrographs were obtained on a SU5000 SEM (Hitachi High‐Technologies) operating with high vacuum at 7 kV accelerating voltage, observation mode (spot size 30) and a BSE detector.

## RESULTS

### qRt‐PCR Detection of SARS‐CoV‐2 in different oral samples

The real‐time RT‐PCR performed on nucleic acids extracted from dental plaque, GCF and saliva samples detected the SARS‐CoV‐2 target genes, with Ct values ranging between 28 and 30. The Ct values obtained for each sample are presented in Table 2. Genotyping tests identified the Delta variant for all three patients. This result confirms the presence of SARS‐CoV‐2 RNA in all oral samples collected from the three patients. The real‐time RT‐PCR were negative for the control negative patient.

**TABLE 2:** Ct values of the *E* gene from oral samples. ‐, no sample. Ct are based on a single determination as samples were tested as part of routine diagnostic RT‐PCR

	**Ct value**		
	Saliva	Dental plaque	GCF
Patient 1	29.55	27.91	29.24
Patient 2	30.25	28.52	28.37
Patient 3	30.96	‐	‐

### FTD respiratory pathogens 21 assay

The real‐time multiplex reverse transcription (RT)‐PCR performed on nucleic acids extracted from dental plaque, GCF and saliva samples were negative for all tested pathogens in the three Sars‐CoV‐2 patients and the negative patient.

### Scanning electron microscopy of dental plaque, GCF and saliva

Oral mucosa cells were identified in different samples based on literature [16, 17]. Large cells resembling non‐keratinized epithelial oral cells possessed free or contacting each other, wavy cell borders and cytoplasm filled with filaments (Figures S1 and S2). A fewer keratinized epithelial oral cell types were also observed, as well as bacteria, generally organized in niches close to keratinized material (Figures S1 and S2).

**FIGURE 1:**
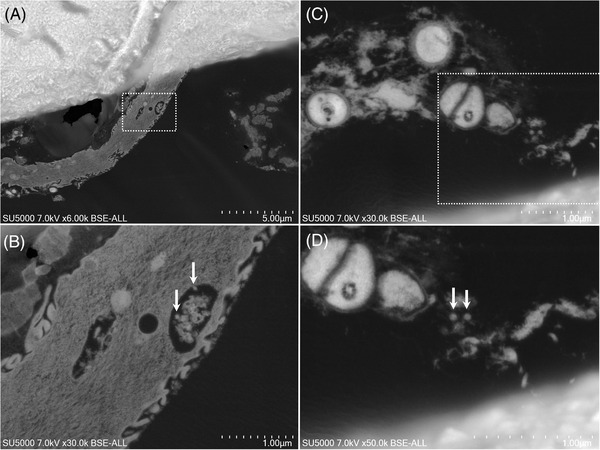
Scanning electron microscopy images of the ultra‐thin section of dental plaque sample. **(A,B)** SEM images of dental plaque sampled from Participant 1. **(B)** High magnification of the boxed region in **(A)** showing electron dense circular structures with 80–140 nm diameters resembled SARS‐CoV‐2‐like particles (arrows) within vesicles at the cell periphery. **(C,D)** SEM images of dental plaque sampled from Participant 2. **(D)** High magnification of the boxed regions in **(D)** showed SARS‐CoV‐2‐like particles (arrows) with 75–140 nm diameters (arrows) (scale bar A: 5 μm; scale bar B‐D: 1 μm).

**FIGURE 2:**
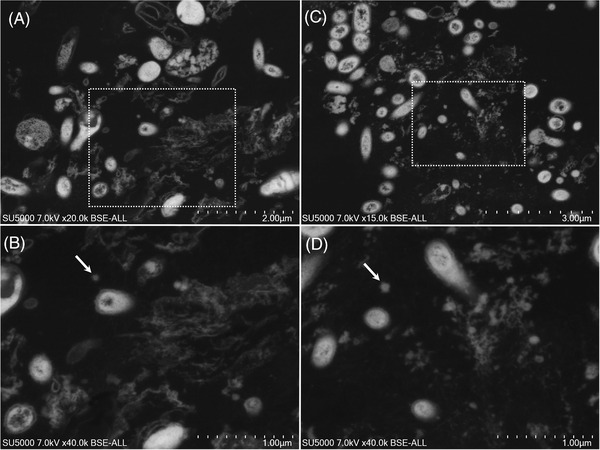
Scanning electron microscopy images of the ultra‐thin section of gingival crevicular fluid sample. **(A,B)** SEM images of (GCF) sampled from Participant 1. **(B)** High magnification of the boxed region in **(A)** showed SARS‐CoV‐2‐like particles (arrows) within vesicles at the cell periphery. **(C,D)** SEM images of (GCF) sampled from Participant 2. **(D)** High magnification of the boxed region in **(D)** showed SARS‐CoV‐2 like particles (arrows) with 75–140 nm diameters (arrows) (scale bar A: 5 μm; scale bar B, C and D: 1 μm) (scale bar A: 2 μm; scale bar B and D: 1 μm; scale bar C: 3 μm).

**FIGURE 3:**
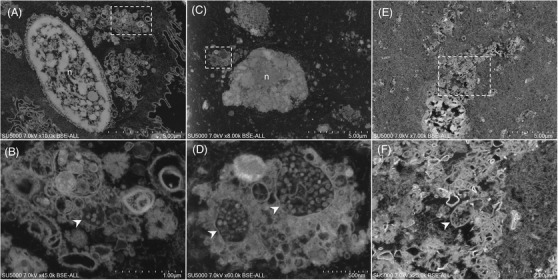
Scanning electron microscopy of saliva sample. **(A,B)** Different magnification views of epithelial oral cell observed in saliva sample of (Participant 1). **(A)** Low magnification of the epithelial oral cell. Boxed regions represent an extensive peri‐nuclear membrane whorls network with vacuoles and (N) nucleus. **(B)** High magnification view of the boxed region in **(A)** showed SARS‐CoV‐2‐like particles inside and outside the closed sac resembling morphogenetic matrix vesicae (arrowhead). **(C,D)** Scanning electron microscopy of epithelial oral cells observed in the saliva sample of Participant 2. **(C)** Low magnification of the epithelial oral cell. Boxed region showed a peri‐nuclear membrane whorls network with vacuoles resembling morphogenetic matrix vesicae, (N) nucleus. **(D)** High magnification view of the boxed region in **(C)** shows SARS‐CoV‐2‐like particles inside open or closed sac with different sizes (arrowhead). **(E,F)** Scanning electron microscopy of the saliva sample of Participant 3. **(E)** Low magnification of oral cell cytoplasm. **(F)** High magnification of the boxed region in **(E)** shows an extensive network membrane with a closed sac containing SARS‐CoV‐2‐like particles (scale bar A, C and E: 5 μm; scale bar B: 1 μm; scale bar D: 500 nm; scale bar F: 2 μm).

In dental plaque (Figure 1) and GCF (Figure 2) samples, electron‐dense circular structures with 75–140 nm diameters resembled SARS‐CoV‐2‐like particles within vesicles at the cell periphery. In saliva samples, several vacuoles dispersed in non‐keratinized cells cytoplasm or at peri‐nuclear locations were observed. These vacuoles, resembled virus morphogenetic matrix vesicae or virions‐rich exit vacuoles of Sars‐CoV‐2 infected cells [18, 19] (Figure 3). These vacuoles were observed (i) empty, or (ii) containing electron‐dense material or filled with particles of different sizes (Figure 3B,D,F). These particle observations in the three samples coincided with the morphology, size and electron density of the imaged particles based on the published description of SARS‐CoV‐2 particles [18, 20, 21]. No such particles were observed in dental plaque (Figure S3), GCF (Figure S4) and saliva (Figure S5) from the negative control patient.

### Immunohistochemistry and correlative light and electron microscopy of dental plaque

**FIGURE 4:**
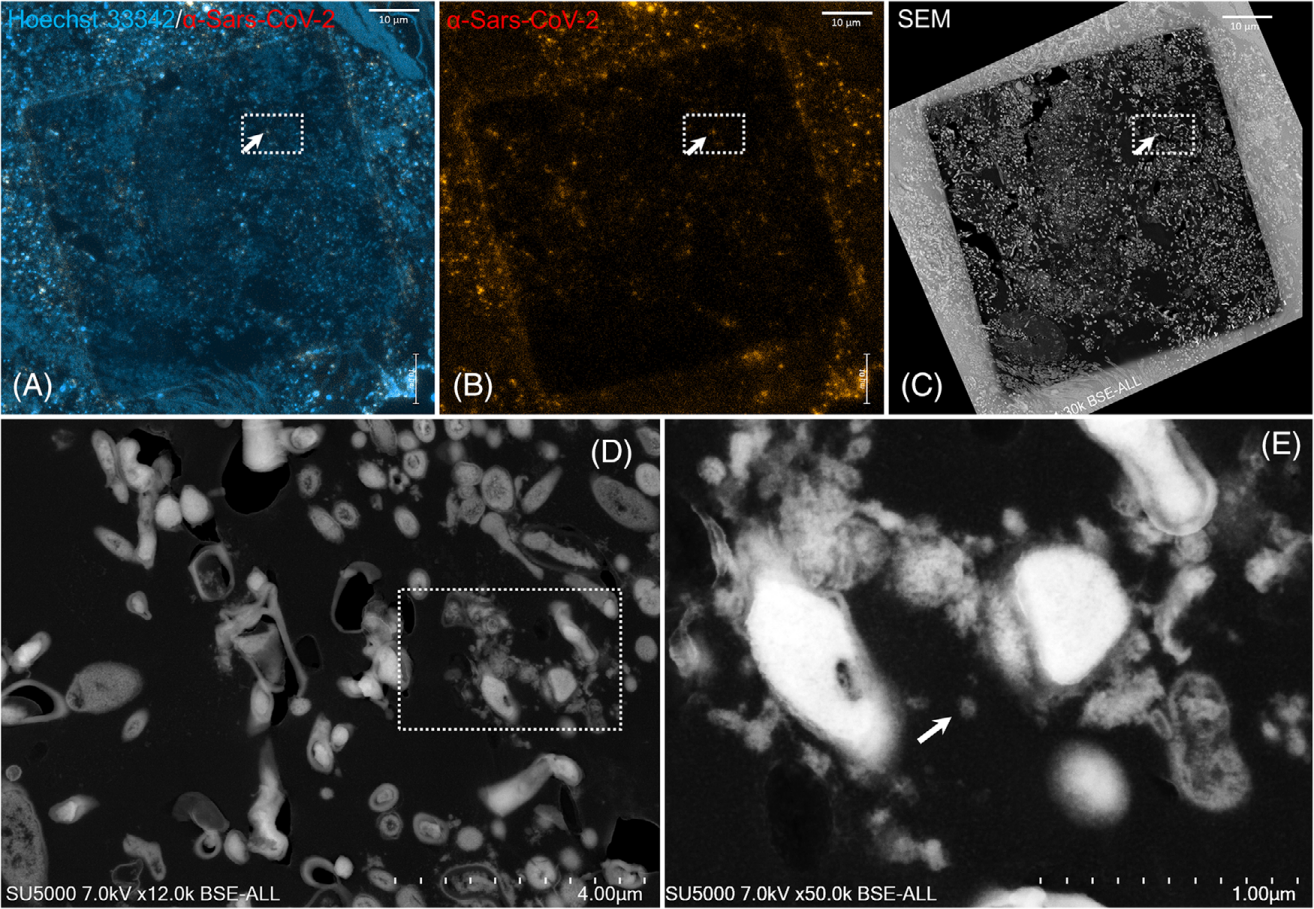
Correlative light fluorescence and electron microscopy in an ultra‐thin section of dental plaque sample (Participant 1). Confocal laser scanning microscopy images of 100 nm‐thick ultra‐thin section (Z maximal projection) of dental plaque sample **(A,B)** DNA stained with Hoechst 33342 (blue) and the SARS‐CoV‐2 particles labelled with anti‐SARS‐CoV‐2 spike protein (orange red). Scanning electron microscopy images **(C–E)** of the ultra‐thin section shown in **(A,B)**. The boxed region of interest in **(A–C)** is shown at a higher magnification in **(D)**. Boxed region in **(D)** is zoomed in **(E)**. Hypo‐electron dense circular structures surrounded by a hyper‐dense crown‐like shapes with 75–140 nm diameters (arrows) are present in the boxed region positive for anti‐SARS‐CoV‐2 fluorescence (scale bar A, B and C: 10 μm; scale bar D: 4 μm; scale bar E: 1 μm).

The immunohistochemistry and correlative light and electron microscopy were applied to ultrathin sections of dental plaque samples. Regions of interest enriched in biological material were identified with the help of Hoechst Dye staining (Figures 4A and S6). Laser‐scanning of the same regions for excitation of the secondary antibody directed against the anti‐SARS‐CoV‐2 antibody was then performed and revealed scattered fluorescent spots among the material in the dental samples (Figures 4B and S6). Regions of interest with positive anti‐SARS‐CoV‐2 fluorescent spots in the ultrathin sections previously acquired by Confocal Laser Scanning Microscopy (CLSM) were scanned a second time by electron microscopy after heavy metal contrast. While scanning the sections using SEM, DAPI staining images were conveniently used as a reference for finding regions of interest containing SARS‐CoV‐2‐positive spots. As seen using SEM, anti‐SARS‐CoV‐2 fluorescence spots were localized in regions containing SARS‐CoV‐2 like particles (Figures 4C–E and S6). The dental plaque sample from the negative control patient was negative for SARS‐CoV‐2 by correlative microscopy (Figure S7). For saliva samples, correlative microscopy could not be performed due to the use of Epon resin, which does not allow immunofluorescence to be performed on the ultra‐thin sections. Correlative microscopy was also not possible for the CGF samples because the amount of material collected was too limited.

### Immunofluorescence assay to detect Sars‐CoV‐2 in saliva samples

**FIGURE 5:**
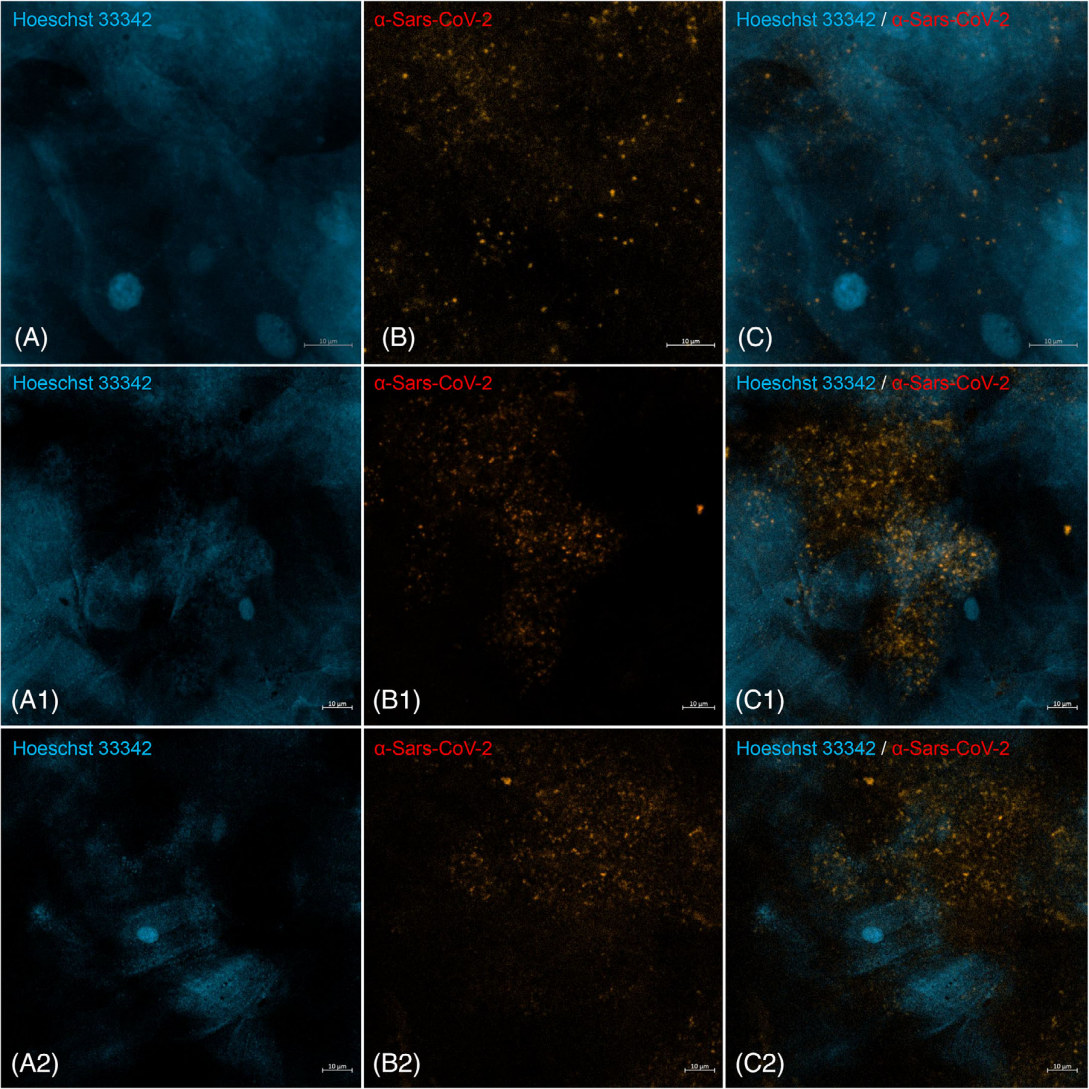
Anti‐SARS‐CoV‐2 immunofluorescence staining on saliva sample from Participant 1 **(A‐C)**, Participant 2 **(A1‐C1)** and Participant 3 **(A2‐C2)**. **(A, A1, A2)** DNA was stained using Hoechst 3342 (blue). **(B, B1, B2)** SARS‐CoV‐2 particles were stained using anti‐SARS‐CoV‐2 antibody (orange‐red), respectively. **(C, C1, C2)** Colocalization of Hoechst 3342 and anti‐SARS‐CoV‐2 antibody (scale bar: 10 μm).

Immunofluorescence staining on saliva samples showed a fluorescence signal of the anti‐SARS‐CoV‐2 antibody inside the epithelial cells (Figure 5), which confirmed the electron microscopy findings. In the saliva sample from the negative control patient, no SARS‐CoV‐2‐like particles were detected in the oral cells and the anti‐SARS‐CoV‐2 immunofluorescence assay was negative (Figure S8).

## DISCUSSION

In this study, using scanning electron microscopy, we observed SARS‐like particles in RT‐qPCR SARS‐CoV‐2‐positive dental plaque, GCF and saliva samples. In addition, the real‐time multiplex reverse transcription (RT)‐PCR performed on dental plaque, GCF and saliva samples were negative for 17 viruses including coronaviruses that could confound our observation with their morphology similar to SARS‐CoV‐2. This finding strongly suggests that the particles observed in the samples could be SARS‐CoV‐2 particles. Furthermore, immunocytochemistry and correlative light‐electron microscopy and immunofluorescence supported these results for dental plaque and saliva samples, respectively.

In this study, we report the direct observation of SARS‐CoV‐2 ‐like particles inside oral mucosa epithelial cells of saliva samples [16, 17]. These findings support the fact that saliva is a potential reservoir of SARS‐CoV‐2 and a potential niche for its replication. ACE2 and TMPRSS2 expressions were detected in the salivary glands [22, 23]. Huang et al. [10] detected SARS‐CoV‐2 in 57% of salivary glands from COVID‐19 autopsy tissues, higher viral loads being found in minor salivary glands, and observed infected acini and ducts that harbored replicating SARS‐CoV‐2. The fact that, in this study, the saliva of an edentulous patient without dentures was positive for SARS‐CoV‐2 might support this result, as no periodontal niche could be involved in the virus load. Huang et al. [10] suggested two potential sources of SARS‐CoV‐2 in the saliva: an acellular fraction from infected glands producing de novo virus and a cellular fraction from infected and shed oral mucosa. The authors also demonstrated the infectiousness of saliva from asymptomatic subjects with COVID‐19. Moreover, in symptomatic patients, the presence of SARS‐CoV‐2 RNA in the saliva was positively associated with the loss of taste and smell [10]. Taking into account all these data and the non‐invasive nature of saliva sampling, saliva seems to be a reliable tool for detecting SARS‐CoV‐2 [24, 25].

Regarding dental plaque, only one study has investigated the presence of SARS‐CoV‐2 RNA in dental biofilms and reported that 18.6% of symptomatic subjects whose nasopharyngeal and oropharyngeal samples tested positive harbored SARS‐CoV‐2 in dental plaque samples [11]. In this study, we confirmed using RT‐PCR that dental plaque samples hosted SARS‐CoV‐2 and we observed viral particles in dental plaque by scanning electron microscopy. As the biofilm formation cycle ends with a detachment phase, during which microorganisms can colonize other surfaces and tissues of the organism [26], dental plaque could play a significant role in viral infection and dissemination. Moreover, periodontal lesions with high viral loads may be at risk of shedding infectious virions which may enter the general circulation [27].

In this study, the presence of SARS‐CoV‐2 RNA in GCF collected from the two study participants and its absence in GCF from the negative control patient were shown by real‐time RT‐PCR while real‐time multiplex reverse transcription (RT)‐PCR that targets 17 viruses including coronaviruses was negative for all samples tested. However, we failed to perform correlative microscopy on GCF due to the limited amount of samples. Therefore, we cannot affirm that particles observed in GCF samples by scanning electron microscopy are SARS‐CoV‐2 particles or other viruses. Thus, improvements in the use of the GCF will need to be made in future work, particularly with regard to the sampling method in order to maximize the quantity of sample taken.

Gingival sulcus and periodontal pockets have recently been suggested as a potential niche for SARS‐CoV‐2 virus infection [28, 29]. This is supported by the fact that several viruses have been detected in GCF [30, 31], that periodontal pockets have been described as a compatible environment for viral infection and survival, and that gingival and periodontal ligament fibroblasts in rat and human tissues could express ACE2, which is considered as the main receptor for the virus entry into target cells [32]. Another hypothesis which could also highlight the relationship between the periodontal pocket and SARS‐CoV‐2 would be that cytokine responses would be common, and the increased cytokines observed in periodontal pockets could exacerbate the COVID‐19‐induced destruction of the lungs [33]. In one recent study, *E* genes of SARS‐CoV‐2 were detected in 63.64% of GCF samples from COVID‐19 positive patients tested by nasopharyngeal swab [12]. This study encourages these findings by the direct observation of SARS‐CoV‐2‐like structures in RT‐PCR positive GCF samples with scanning electron microscopy. GCF is a physiological fluid, as well as an inflammatory exudate, which has been extensively explored in the search for potential diagnostic biomarkers of periodontal disease [34, 35]. However, we concluded in this study that GCF did not appear to be the most easily usable type of sample for the observation of virus particles. Saliva and dental plaque samples provided better results and thus seem to be more easily exploitable samples in clinical practice and for systematic use on a larger scale. The presence of SARS‐CoV‐2 in dental plaque and saliva samples supports the hypothesis that the oral cavity may be defined as a potential reservoir for this virus. Thus, expelled oral droplets carrying infectious virus and infected cells, notably expelled during dental care, are a potential risk for the airborne transmission of SARS‐CoV‐2. SARS‐CoV‐2 was detected in aerosols during ultrasonic scaling and tooth preparation with medium‐volume suction [36]. Protective devices have been described to reduce aerosol dispersion and thus, the risk of contamination, such as rigid translucent acrylic structure covering the patient's head, neck and chest, the use of high‐speed suction and the use of preoperative antimicrobial mouth rinses [37, 38].

Nevertheless, the results herein should be considered in the light of some limitations. Only three study participants with different periodontal conditions and one control were included. Further studies including a larger number of patients would be of interest to confirm these preliminary results. In addition, this study is a cross‐sectional study and therefore does not provide information on the evolution of the presence of SarS‐CoV‐2 particles over time. Longitudinal sampling could add significant additional knowledge.

In conclusion, the direct observation of SARS‐CoV‐2‐like particles in different oral samples in this study supports the results of previous studies and confirms that the oral cavity may be a potential niche for SARS‐CoV‐2 infection. Minimally invasive screening and diagnostic tools for COVID‐19 using these samples should be investigated in further studies.

## CONFLICT OF INTEREST

The authors declare that the research was conducted in the absence of any commercial or financial relationships that could be construed as a potential conflict of interest.

## AUTHOR CONTRIBUTIONS

**Conceptualization**: D Brahim Belhaouari, J‐P Baudoin, B La Scola, A Antezack. **Methodology**: D Brahim Belhaouari, J‐P Baudoin, B La Scola, A Antezack. **Formal analysis**: D Brahim Belhaouari, J‐P Baudoin. **Investigation**: D Brahim Belhaouari, J‐P Baudoin, A Antezack. **Writing – original draft preparation**: D Brahim Belhaouari, A Antezack. **Writing – review and editing**: J‐P Baudoin, J‐C Lagier, B La Scola, V Monnet‐Corti.

## FUNDING

This work was supported by the French Government under the “Investissements d'avenir” programme managed by the National Agency for Research (ANR), Méditerranée‐Infection 10‐IAHU‐03. In addition, collaborative work conducted by IHU Méditerranée Infection and the Hitachi High‐Tech Corporation is funded by the Hitachi High Tech Corporation.

## Supporting information

Supporting Information
