# Supplementary material for: Microscopic observations of SARS‐CoV‐2 like particles in different oral samples
Source: Eur J Oral Sci. 2022 Nov 20;130(6):e12903. doi: 10.1111/eos.12903 (PMC10099536; doi:10.1111/eos.12903)
Supplement: Supplementary file 1 — Supporting Information [file EOS-130-0-s001.pdf]

SUPPORTING INFORMATION

Microscopic observations of SARS-CoV-2 like particles in different oral samples

DJAMAL BRAHIM BELHAOUARI, JEAN-PIERRE BAUDOIN, JEAN-CHRISTOPHE LAGIER, VIRGINIE MONNET-CORTI, BERNARD LA SCOLA, ANGELINE ANTEZACK

Institut de Recherche Pour Le Développement (IRD), Aix-Marseille Université, Provence-Alpes-Côte D'azur, Marseille, France.

Ecole de Médecine Dentaire, Aix-Marseille Université, Provence-Alpes-Côte D'azur, Marseille, France.

Hopital Timone, Provence-Alpes-Côte D'azur, Marseille, France

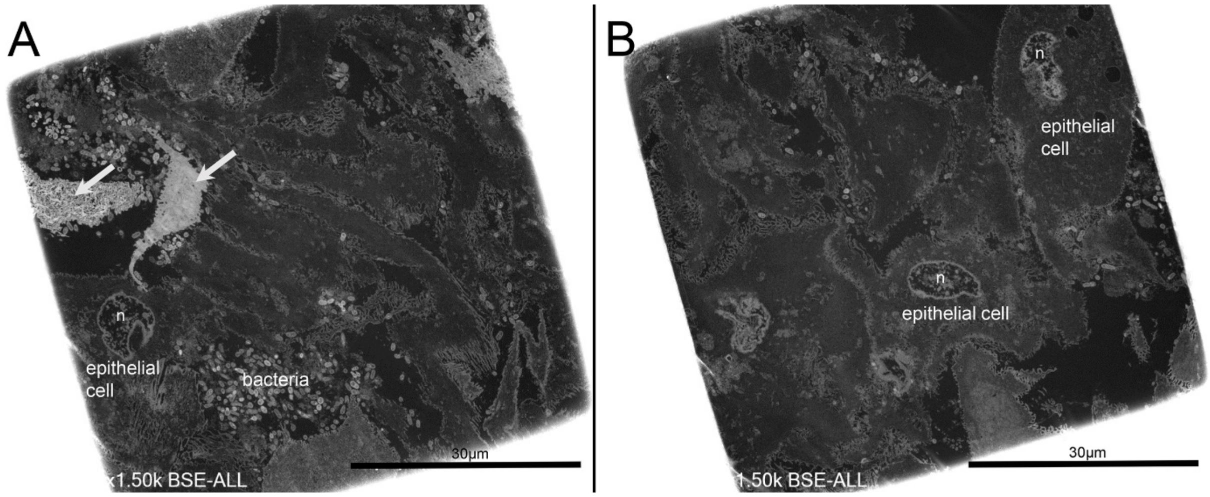

Figure S1. Scanning electron microscopy images **(A,B)** showing large cells resembling non-keratinized epithelial oral cells. (n): nucleus, arrows correspond to material resembling keratinized epithelial cells. Bacteria were observed generally organized in niches close to the cells (scale bar: 30µm).

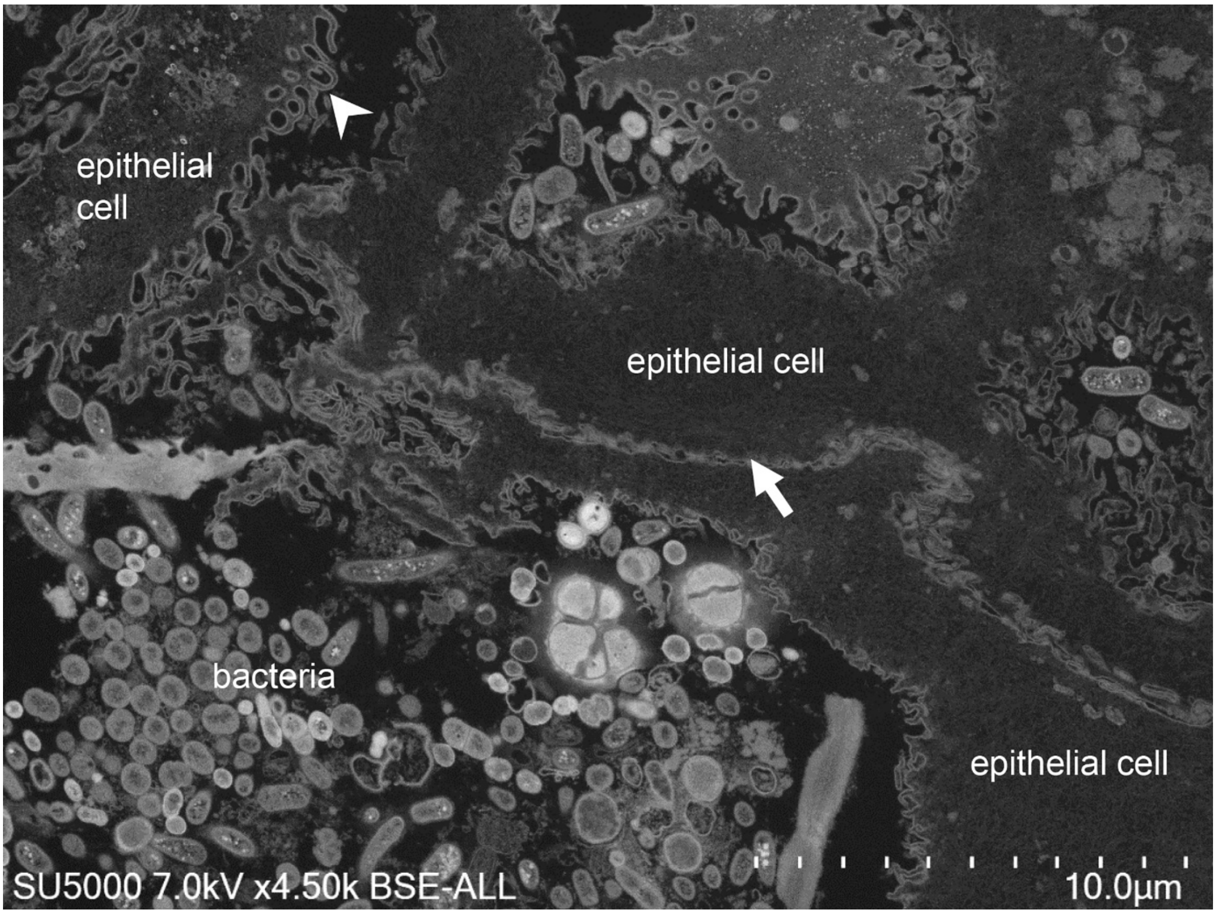

Figure S2. Scanning electron microscopy images showing epithelial oral cells possessed free (arrowhead) or contacting each other (arrow) (scale bar: 10µm).

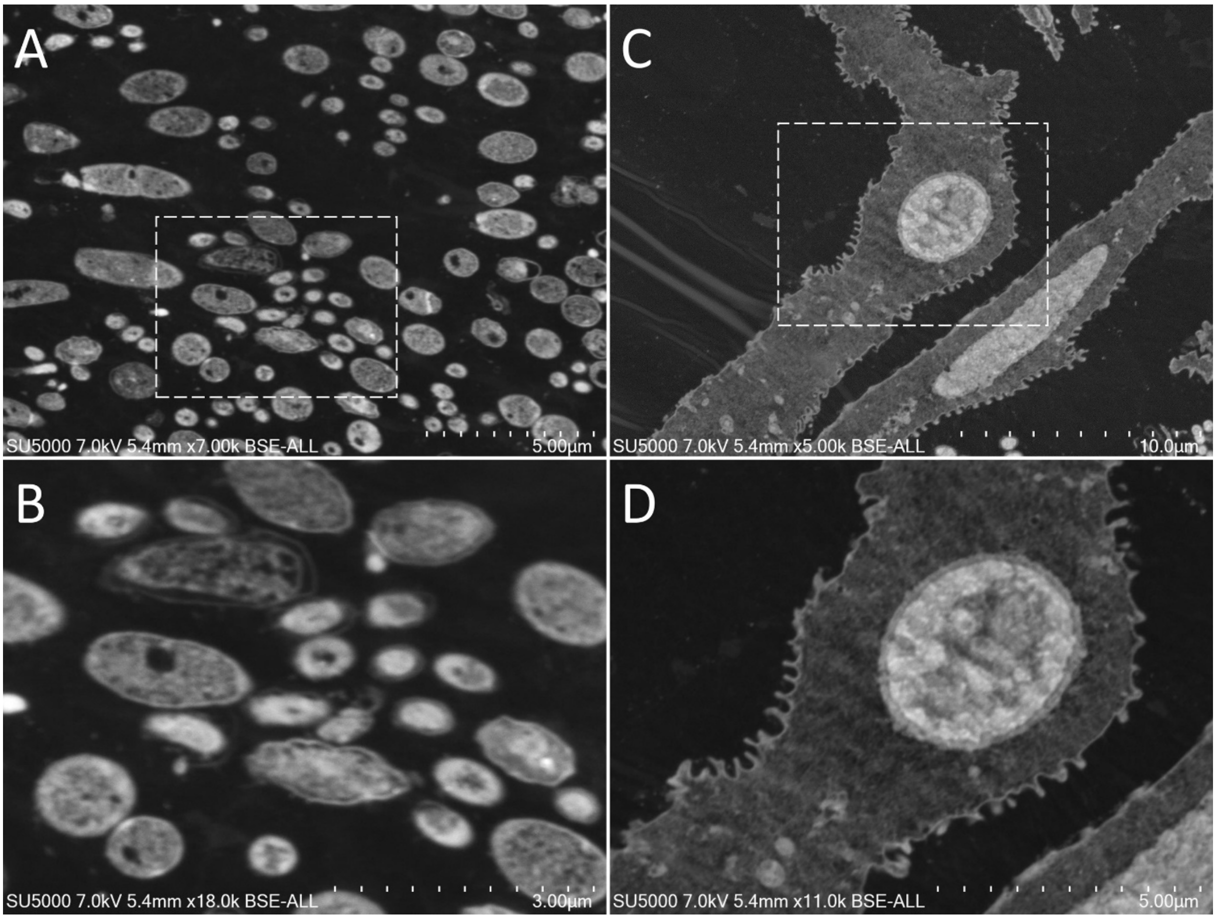

Figure S3. Scanning electron microscopy of dental plaque sample of negative control patient. **(A)** Overview of image of different microorganism ultrastructures observed on the dental plaque (scale bar: 5µm). **(B)** High magnification view of the boxed region in **(A)** showing the absence of SARS-CoV-2-like ultrastructure particles (scale bar: 3µm). **(C)** Overview of epithelial oral cells observed in dental plaque sample of negative control patient (scale bar: 10µm). **(D)** High magnification view of the boxed region in **(C)** showing the absence of morphogenetic features and infection of SARS-CoV-2 in the cytoplasm and precisely in the perinuclear region (scale bar: 5µm).

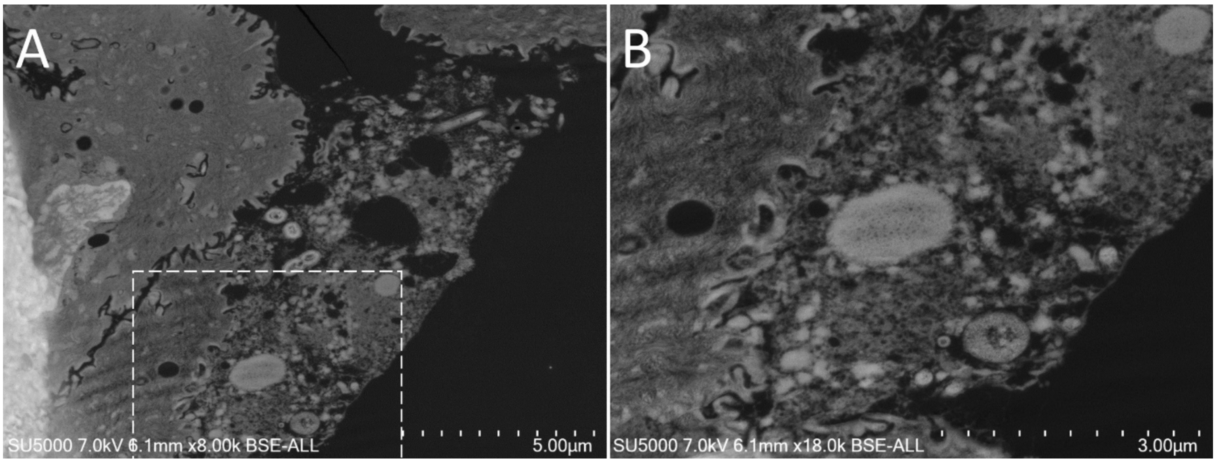

Figure S4. Scanning electron microscopy of GCF sample of negative control patient. **(A)** Overview of oral cell observed in GCF (scale bar: 5μm). **(B)** High magnification view of the boxed region in **(A)** showed the absence of morphogenetic features and infection of SARS-CoV-2 (scale bar: 3μm).

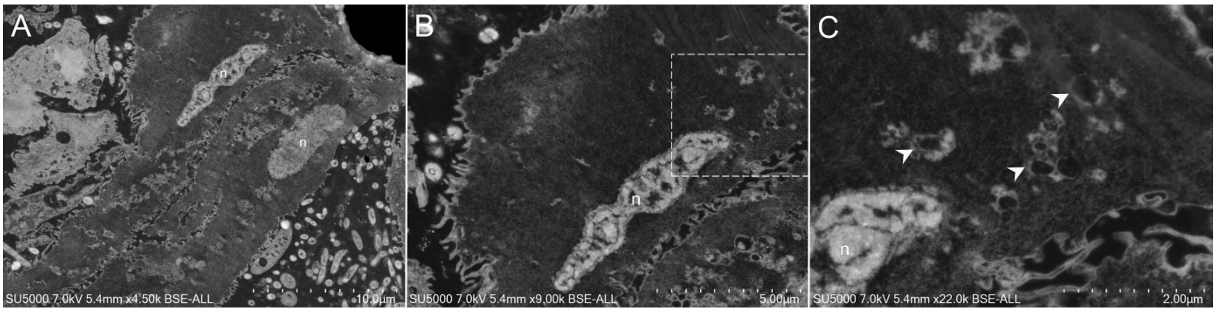

Figure S5. Scanning electron microscopy of saliva sample of negative control patient. **(A)** Overview of oral cell observed in saliva (scale bar: 10μm). **(B)** Low magnification of the oral cell (scale bar: 5μm). **(C)** High magnification view of the boxed region in **(B)** showed several vesicles dispersed in the cytoplasm of the cell and without SARS-CoV-2-like particles inside (scale bar: 2μm).

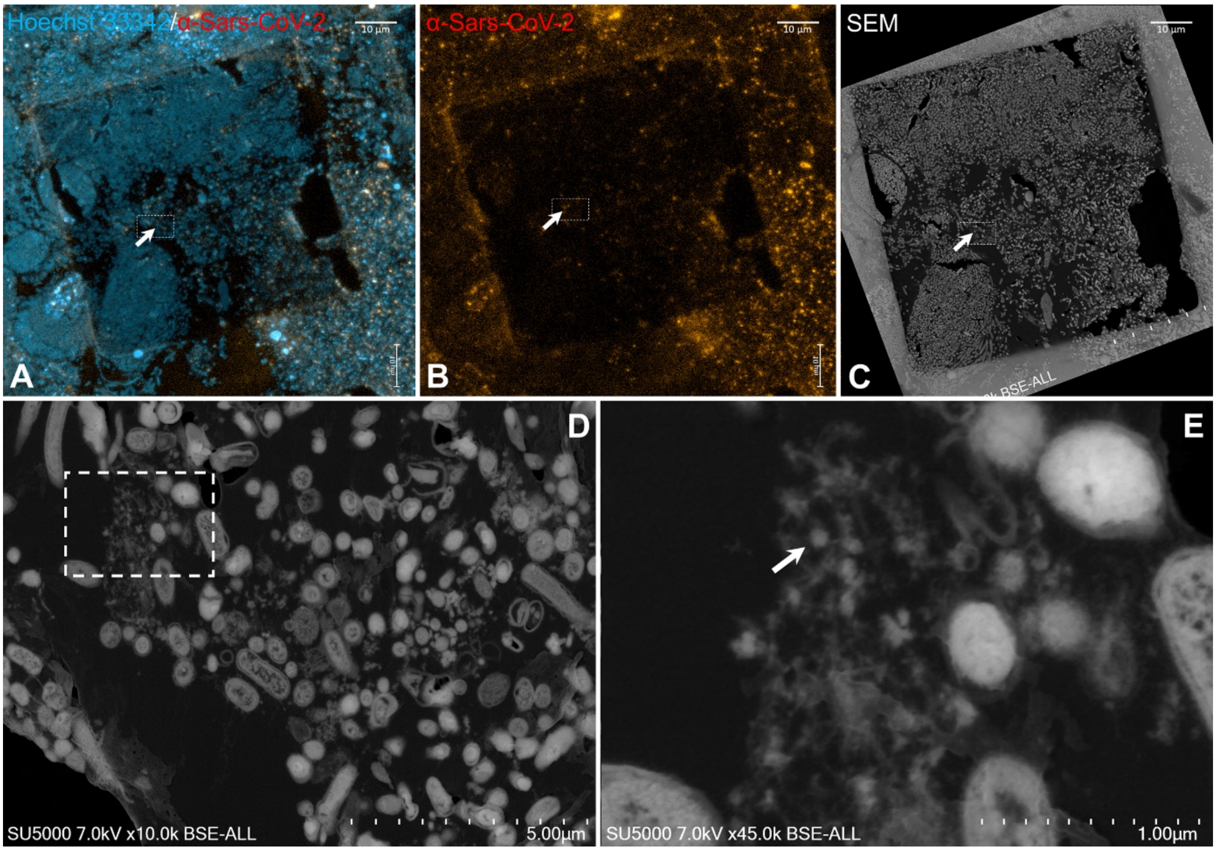

Figure S6. Correlative light fluorescence and electron microscopy in an ultra-thin section of dental plaque sample from Patient 2. Confocal laser scanning microscopy images of 100 nm-thick ultra-thin section (Z maximal projection) of the dental plaque sample **(A, B)**. DNA stained with Hoechst 33342 (blue) and the SARS-CoV-2 particles labelled with anti-SARS-CoV-2 spike protein (orange red). Scanning electron microscopy images **(C–E)** of the ultra-thin section shown in **(A, B)**. The boxed region of interest in **(A–C)** (scale bar: 10µm) is shown at a higher magnification in **(D)** (scale bar: 5µm). Boxed region in **(D)** is zoomed in. **(E)** Hypo-electron dense circular structures surrounded by a hyper-dense crown-like shapes with 75–140 nm diameters (arrows) are present in the boxed region positive for anti-SARS-CoV-2 fluorescence (scale bar: 1µm).

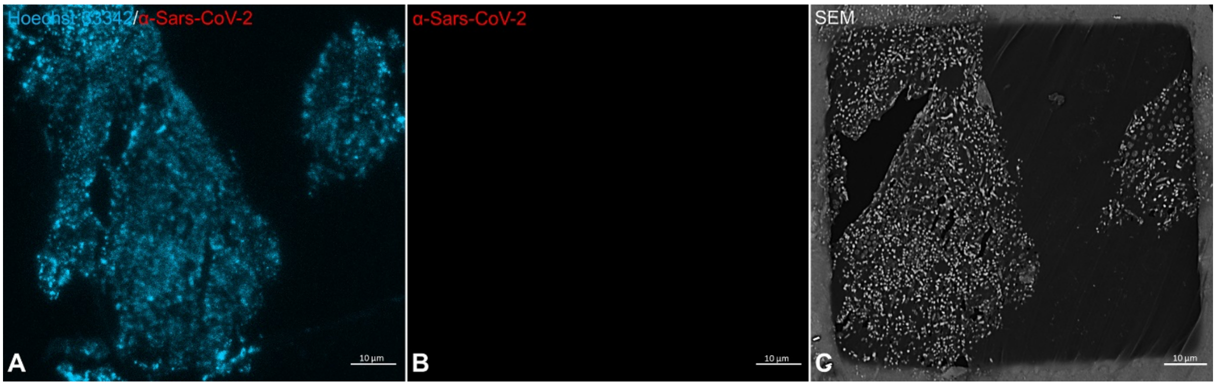

Figure S7. Correlative light fluorescence and electron microscopy in an ultra-thin section of dental plaque sample of negative control patient. **(A, B)** Confocal laser scanning microscopy images of 100 nm-thick ultra-thin section (Z maximal projection) of dental plaque sample

stained with Hoechst 33342 (blue) and with anti-SARS-CoV-2 spike. **(C)** Scanning electron microscopy image of the ultra-thin section shown in **(A, B)** (scale bar: 10µm)

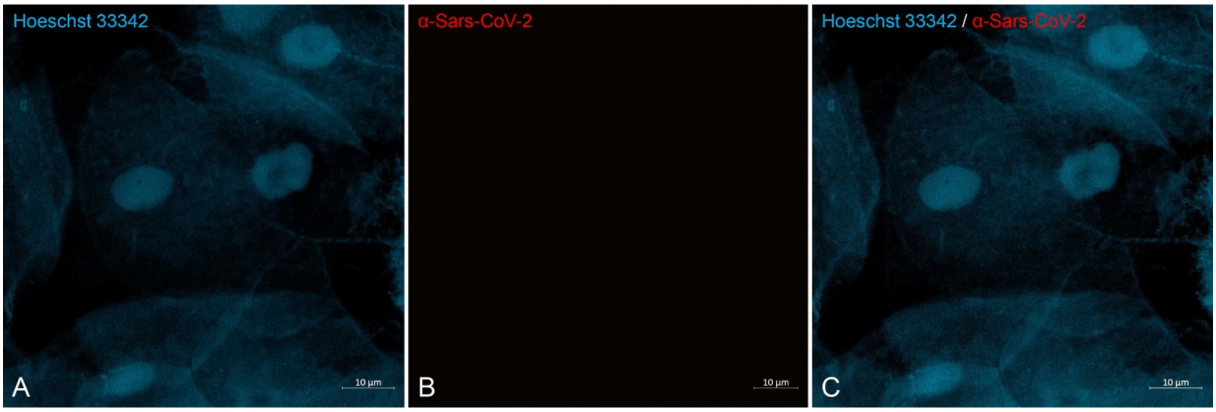

Figure S8. Anti-SARS-CoV-2 immunofluorescence staining on saliva sample of negative control patient. **(A)** DNA was stained using Hoechst 3342 (blue). **(C)** Immunofluorescence staining for SARS-CoV-2 did not show a fluorescence signal colocalization of **(A)** and **(B)** (scale bar: 10µm)
